# Spermine oxidase (SMO) activity in breast tumor tissues and biochemical analysis of the anticancer spermine analogues BENSpm and CPENSpm

**DOI:** 10.1186/1471-2407-10-555

**Published:** 2010-10-14

**Authors:** Manuela Cervelli, Gabriella Bellavia, Emiliano Fratini, Roberto Amendola, Fabio Polticelli, Marco Barba, Rodolfo Federico, Fabrizio Signore, Giacomo Gucciardo, Rosalba Grillo, Patrick M Woster, Robert A Casero, Paolo Mariottini

**Affiliations:** 1 Dipartimento di Biologia, Università Roma Tre, Rome, Italy; 2 Dipartimento BAS-BiotecMed, ENEA, CR Casaccia, Rome, Italy; 3 Department of Gynaecology, Breast Surgery and Pathology, San Camillo-Forlanini Hospital, Rome, Italy; 4 Department of Pharmaceutical Sciences, Wayne State University, Detroit, MI 48202, USA; 5 The Sidney Kimmel Comprehensive Cancer Center at Johns Hopkins, The Johns Hopkins University School of Medicine, Baltimore, MD 21231, USA

## Abstract

**Background:**

Polyamine metabolism has a critical role in cell death and proliferation representing a potential target for intervention in breast cancer (BC). This study investigates the expression of spermine oxidase (SMO) and its prognostic significance in BC. Biochemical analysis of Spm analogues BENSpm and CPENSpm, utilized in anticancer therapy, was also carried out to test their property *in silico *and *in vitro *on the recombinant SMO enzyme.

**Methods:**

BC tissue samples were analyzed for SMO transcript level and SMO activity. Student's t test was applied to evaluate the significance of the differences in value observed in T and NT samples. The structure modeling analysis of BENSpm and CPENSpm complexes formed with the SMO enzyme and their inhibitory activity, assayed by *in vitro *experiments, were examined.

**Results:**

Both the expression level of SMO mRNA and SMO enzyme activity were significantly lower in BC samples compared to NT samples. The modeling of BENSpm and CPENSpm complexes formed with SMO and their inhibition properties showed that both were good inhibitors.

**Conclusions:**

This study shows that underexpression of SMO is a negative marker in BC. The SMO induction is a remarkable chemotherapeutical target. The BENSpm and CPENSpm are efficient SMO inhibitors. The inhibition properties shown by these analogues could explain their poor positive outcomes in Phases I and II of clinical trials.

## Background

Breast cancer (BC) is a common disease that generally occurs in women over the age of 50, and the risk is especially high for women over age 60. Patients who undergo curative surgery may develop metastasis during follow-up, and the side effects of cancer treatment depend mainly on the type and extent of the treatment. One of the major therapeutic problems is that tumors initially responsive to chemotherapeutic approaches can progress to more aggressive forms poorly responsive to therapies. The need for antineoplastic compounds with novel mechanisms of action is therefore of high social impact. The polyamines (PA) are polycations essential for cell growth and differentiation [1]. In BC cells, proliferative signals transduced by estradiol and growth factors are modulated by PA, by the induction of ornithine decarboxylase (ODC) [2,3]. Increased PA levels are often associated with malignant transformation and maintenance of the neoplastic phenotype [4]. Cells finely regulate PA concentrations by *de novo *synthesis from amino acid precursors and PA uptake from diet, with the balancing inter-conversion, stepwise degradation and efflux. In the last decade PA metabolism has been studied in detail and the enzymes involved in the PA biosynthesis and catabolism well characterized [5,6]. PA facilitate the interactions of transcription factors, such as estrogen receptors and nuclear factor kB, with their specific response element [7] and are also involved in the proliferation of ER-negative and highly invasive models of tumor cells [8]. Consequently, PA pathway is an important target for drug development for BC [9]. A recent strategy in anticancer therapy is to exploit the self-regulatory nature of PA metabolism through the use of PA analogues to affect PA homeostasis [10]. The importance of the PA catabolic pathway has been re-evaluated [5,6], since its involvement in determining the cell response to antitumor PA analogues has been demonstrated [11]. An analysis of spermidine/spermine *N*^1^-acetyltransferase (SSAT) and *N*^1^-acetylpolyamine oxidase (APAO) enzyme activities in human BC tissue has been carried out by Wallace et al. [12]. This analysis correlates the higher level of acetylated polyamines (acetylPA) in malignant tumors, with the decreasing activity of APAO, concurrent with the increase of SSAT activity [12].

To determine the incidence of spermine (Spm) analogues in BC treatments, we previously evaluated the level of spermine oxidase (SMO) expression in BC tissues. Our results point out that SMO enzyme activity, characterized by a Spm substrate specificity, is significantly lower in BC than in healthy tissues.

Among the Spm analogues, bis(ethyl)norspermine (BENSpm) has been well characterized and underwent Phase I and II clinical trials [13,14]. The antiproliferative effects of BENSpm on some human BC cell lines, like MDA-MB-231 cells, seem to be mediated in part through the production of H_2_O_2 _by SMO and by the export of acetylPA formed by the induction of SSAT activity [11]. Results from Phase II study of therapeutic use of BENSpm against advanced refractory BC revealed that BENSpm was not active as a single agent [14]. Extension of this study has been recently carried out to demonstrate the ability of BENSpm to synergize with other standard chemotherapeutic agents on the treatment of some human BC cell lines [15]. The second generation Spm analogue N**^1^**-ethyl-N**^11^**-(cyclopropyl)-methyl-4,8-diazaundecane (CPENSpm) [16] has demonstrated lower toxicity and greater therapeutic efficacy than the first generation compounds. CPENSpm has been used in combination with other cytotoxic drugs in the treatment of BC cell lines, but it demonstrates to be cell type specific [17,18]. Although experimental protocols were promising on the clinical use [19], BENSpm Phase I and II clinical trials gave poor positive outcomes [14,18,20,21]. Analogously, the utilization of the CPENSpm has produced results similar to those obtained with the BENSpm treatments [22].

In this framework, we perform a first experimental study to correlate the level of SMO expression in BC tissues and structure modeling analysis of the complexes of BENSpm and CPENSpm formed with the SMO enzyme. Since the cellular mechanism of BENSpm and CPENSpm action is still unclear, we analyze if these Spm analogues are inhibitors of the SMO enzyme [5,6]. Structure modeling analysis of the complexes of BENSpm and CPENSpm formed with the SMO enzyme supported the hypothesis that these analogues could bind in the catalytic site of the SMO protein. The Ki values of BENSpm and CPENSpm have also been measured, revealing that both analogues behave like SMO inhibitors. The inhibitory role of Spm analogues and the low level of SMO in BC tissues taken together, could explain the poor positive outcome of both BENSpm, in Phases I and II of clinical trials, and CPENSpm, due to a lower H_2_O_2 _production inside tumor mass [11].

## Methods

### Patients

Patients were admitted to the Department of Breast Surgery at San Camillo-Forlanini Hospital (Rome, Italy), investigations were carried out in all patients before surgery: mammography and breast ultrasound, breast MRI if requested, breast microbiopsy with Mammotome or Tru-cut tecniques, blood tests (hemoglobin and full blood count, urea and electrolytes, liver function tests), chest radiography. Twenty patients were selected based on primary breast carcinoma that have not been previously treated with chemotherapy or radiotherapy. The size of the breast tumor was measured clinically using the standard tumor size grading system (TNM). Immediately after removal of the tumor from the patients, a tumor (T) sample measuring ~0.5 cm in diameter was removed from the tumor mass, cooled immediately with dry ice, and then stored at -80°C until analyses were undertaken. As a control, a piece of nontumor (NT) breast tissue (1 cm of diameter) was removed as far away as possible from the quadrant of breast containing tumor. NT samples were treated with the same protocol as the T samples.

Before surgical procedure all patients signed an informed medical consent form, previously approved by the Institutional Ethical Committee and the Medical Board of the San Camillo-Forlanini Hospital.

### Histology

The histological type and grade for breast tumors were determined by an author (RG) of the present work, who is an expert breast pathologist.

### PCR analysis

The relative levels of human SMO, APAO, ODC, SSAT, β-actin and glyceraldehyde-3-phosphate dehydrogenase (GADPH) transcripts were measured by semiquantitative RT-PCR with specific primers (Additional file 1: Table S1) as described in Cervelli et al. [23]. Data obtained from single patients were pooled and analyzed to produce an average level of expression. The RT-PCRs were normalized by comparison either with the β-actin or the GADPH controls according to the number of PCR cycles reaction adopted. Further control reaction mixtures, either without template (not shown) or RT enzyme (not shown), were uniformly negative. Results were quantified by densitometry, using the BioRad Multianalyst software (BioRad, Hercules, CA). An estimate of the relative RT-PCR amplified product amounts was obtained by dividing the area of gel bands by the area of the relative control, alternatively β-actin or GADPH gel band. Data points are the means (Standard Deviation, SD) of three to five separate experiments, each performed in duplicate. The p values were measured with the Student's t test.

### Analysis of enzymatic activities

SMO and APAO activities on BC tissue homogenates were determined according to Wang et al. [24]. Two hundred and fifty μg of tissue sample was used for the assay utilizing Spm and N^1^-acetylated Spm as substrates for SMO and APAO, respectively. In particular, SMO and APAO enzymatic activities were determined by measuring the production of H_2_O_2 _as pmol produced/mg protein/h, following the oxidation of their specific substrates. Protein content was estimated by the method of Bradford [25]. In details, enzyme activity was assayed in 83 mM glycine buffer, pH 8.0, 5.0 nmol luminol, 20 μg horseradish peroxidase, 0.2 mM 2-bromoethylamine (copper-containing amine oxidase inhibitor/catalase inhibitor), 15 μM deprenyl (mitochondrial oxidase B inhibitor), 0.15 mM clorgyline (mitochondrial oxidase A inhibitor) and 250 μM Spm or alternatively N^1^-acetylated Spm as the substrate. All reagents with the exception of substrate were combined in a volume of 250 μl and incubated for 2 minutes at 37°C, transferred to the luminometer where substrate was added, and the resulting chemiluminescence was integrated over 40 seconds. Both ODC and SSAT activities were determined as pmol CO_2 _produced/mg protein/h by using ^14^C-labeled substrate and scintillation counting of end metabolized products. BC tissues were sonicated and centrifuged at 22,000 *g *for 10 min at 4°C.

The SSAT enzyme activity was determined as described by Chen et al. [26]. In particular, 75 μg of homogenate BC tissue sample in a final volume of 50 μl included 10 μl of 5.5 M Bicine buffer (pH 8.0), 5 μl of 30 mM Spd, 10 μl of doubly distilled water, 5 μl of 0.1 mM [^14^C]acetyl-CoA (53 mCi/mmol; Sigma) and 20 μl of each sample. The mixture was incubated for 5 min at 37°C. The enzyme reaction was stopped by the addition of 20 μl 0.5 M hydroxylamine hydrochloride, and the mixture was heated in boiling water for 3 min. The resulting samples were centrifuged (22,000 g), and an aliquot of 50 μl was spotted onto Whatman P81 phosphocellulose discs and counted for radioactivity in a liquid-scintillation counter.

The ODC enzyme activity was determined as follows: 100 μg of homogenate BC tissue sample in a final volume of 100 μl including 20 mM Tris Buffer (pH 7.5), 1 mM EDTA, 0.1 mM Piridoxal 5' POH, 5 mM DTT, 0.1 mM [^14^C] L-Ornithine-carboxy (55 mCi/nmol; Sigma) and 0.4 mM L-Ornithine was put in a screwed-cap plastic "bijoux" vial. A disk of filter paper, embedded with 30 μl of 2N NaOH, was screwed with the cap on the top of the vial. The mixture was incubated for 30 min at 37°C. The enzyme reaction was stopped by the addition of 200 μl of 10% TCA, then the mixture was further incubated for 10 min at 37°C to ensure complete CO_2 _adsorption on the filter paper that was finally counted for radioactivity in a liquid-scintillation counter. Statistical differences were analyzed by Wilcoxon matched pairs signed rank test.

### Molecular modeling of mSMO-BENSpm and mSMO-CPENSpm complexes

The molecular models of the complexes formed by MDL 72527 (N^1^, N^4^-bis(2,3-butadienyl)-1,4butanediamine), BENSpm and CPENSpm with mSMO were built using the crystal structure of maize PAO (MPAO) as a template (PDB code: 1B37; [27]). The multiple sequence alignment between mSMO, MPAO and other PAOs was obtained using the program CLUSTALW [28]. Based on this alignment, the three-dimensional structure of mSMO was then built using Modeller [29]. mSMO-Spm complex was then built using the FMS1-Spm complex as a template (PDB code: 1XPQ; [30]) and this complex was used to dock the inhibitors into mSMO active site by superimposition of MDL 72527, BENSpm and CPENSpm onto the Spm moiety.

### Inhibition assays

The SMO activity was assayed at pH 8.5 (1.0 × 10^-1 ^M sodium borate buffer) and at 25°C (SMO enzyme concentration ranging from 2.0 × 10^-8 ^M to 5.0 × 10^-8 ^M). The substrate Spm was used with a concentration ranging from 4 × 10^-6 ^M to 10 × 10^-6 ^M in the presence or absence of BENSpm and CPENSpm. In the enzyme assay the BENSpm ranged between 1.8 × 10^-4 ^M and 7.1 × 10^-4 ^M, while CPENSpm between 9.5 × 10^-5 ^M and 3.7 × 10^-4 ^M. The *K*_i _values were determined according to the Dixon [31] graphical method. Data reported are the average of three different experiments, each with two replicates, standard deviation (SD) was 5%.

## Results

### Patient demographics

A total of 20 patients with a mean age of 72.9 ± 11.8 (SD) years were included in the study. Table 1 details the clinic pathologic variables for each patient.

**Table 1:** Patient demographics

Patient No.	Age year	TNM	Tumoral markers	Tumor grade	Histological type of cancer
1	67	T4 N1A M0	E90%, P90%, K10%, C1+	II	IDC
2	86	T1C N1 M0	E90%, P90%, K15%, C2+	II	IDC
3	80	T1B N0 M0	E90%, P90%, K15%, CNEG	II	IDC
4	71	T1C N0 M0	ENEG, PNEG, K40%, C3+	III	IDC
5	75	T2 N1 M0	ENEG, PNEG, K30%, CNEG	III	IDC
6	62	T1C N0 M0	E90%, P90%, K30%, C2+	II	IDC
7	84	T4 N1 M0	E90%, PNEG, K40%, C3+	III	IDC
8	76	T2 N0 M0	E90%, P90%, K30%, CNEG	III	IDC
9	88	T2 N0 M0	E90%, P90%, K15%, C1+	II	IDC
10	50	T2 N2A M0	E90%, P90%, K20%, C2+	III	IDC
11	85	T1C N1 M0	ENEG, PNEG, K40%, CNEG	III	IDC
12	82	T4 N1 M0	E90%, P40%, K20%, C3+	II	IDC
13	53	T2 N2 M0	E90%, P90%, K30%, CNEG	III	ILC
14	68	T2 N1 M0	E90%, P90%, K20%, C1+	II	IDC
15	66	T2 N0 M0	E90%, P90%, K30%, C2+	III	IDC
16	73	T2 N1 M0	E80%, P70%, K20%, C3+	II	IDC
17	64	T2 N0 M0	E80%, P70%, K30%, C2+	III	IDC
18	63	TIS	ENEG, PNEG, K40%, C3+	III	ISDC
19	47	T2 N0 M0	E90%, P90%, K30%, CNEG	III	IDC
20	70	T1C N0M0	ENEG, PNEG, K40%, C3+	III	IDC

C: c-erb; E: estrogen receptor; K: Ki67; NEG: negative; P: progesteron receptor; TNM: tumor, node, metastasis; IDC: Invasive Ductal Carcinoma; ILC: Invasive Lobular Carcinoma; DCIS: *In situ *Ductal Carcinoma; TIS: *In Situ*Tumor.

### Gene expression and enzymatic activities of SMO, APAO, ODC and SSAT in BC and nonneoplastic breast tissues

**Figure 1:**
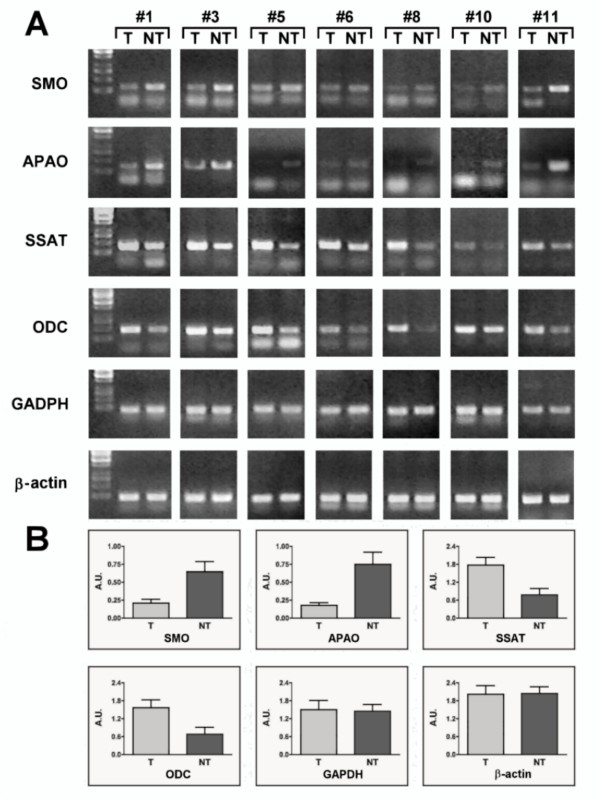
**RT-PCRs determination of selected genes in BC tissues**. Samples from tumor (T) and nontumor (NT) tissues were treated and analyzed as described in Methods. The PCR products were fractionated by 1.2% agarose gel electrophoresis. (A) Representative RT-PCR experiments from three independent replicas are shown. (B) Densitometric analyses of PCR gel bands, obtained from patients, represent the measurements done on three separate experiments. Data were pooled and analyzed to produce an average level of expression. Alternatively, gene of interest/β-actin or/GADPH ratios (normalized for each experimental time point) have been used for normalization. An arbitrary densitometric unit bar graph (SD) is shown. The p values (< 0.01) were measured with the Student's t test.

**Figure 2:**
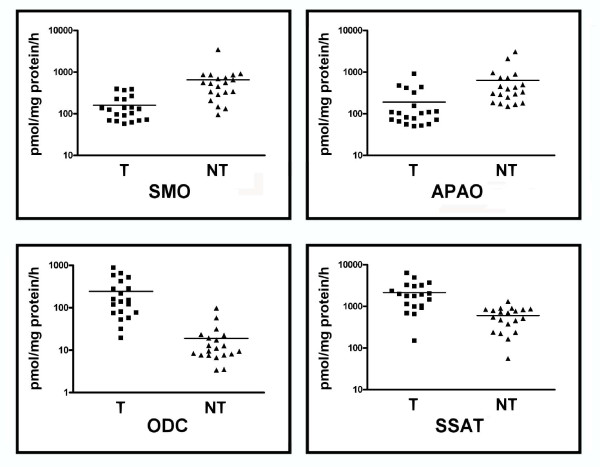
**SMO, APAO, ODC and SSAT activities**. Enzyme activities from tumor (T) and nontumor (NT) samples were assayed as described in Methods section. Results are mean (SD) with n value of 20. The p values (< 0.05) were measured with Wilcoxon matched pairs signed rank test.

Semi-quantitative RT-PCR indicated that the mean expression levels of SMO mRNA are significantly lower (p < 0.01) in T tissue than in NT tissue (Figure 1). Analogously, APAO transcript was confirmed to be lower in tumor samples than in the normal tissue (p < 0.01). On the contrary, ODC and SSAT transcripts show an opposite expression profile (p < 0.01) (Figure 1). The enzymatic activities measured from the same samples paralleled the mRNA level profiles between T and NT tissues (Figure 2). SMO, APAO, ODC and SSAT enzymatic activities measured on each individual sample are reported (Additional file 2: Table S2). The SMO activity was significantly lower (< p 0.05) in tumor tissue compared with the equivalent normal tissue (Figure 2). APAO enzymatic activity was confirmed to be significantly lower (< p 0.05) in T samples than in the NT tissues, by contrast, both ODC and SSAT activities were significantly higher (< p 0.05) in malignant samples than in normal tissue (Table 2).

**Table 2:** Enzyme activities in human BC tissues

S	N	SMO activity (pmolH_2_O_2_/mgprotein/h)	APAO activity (pmolH_2_O_2_/mg protein/h)	ODC activity (pmolCO_2_/mg protein/h)	SSAT activity (pmolCO_2_/mg protein/h)
T	20	159.1 (024.8)	190.8 (048.9)	244.5 (53.7)	2,150.0 (343.0)
NT	20	653.2 (161.1)	631.4 (164.1	19.0 (05.2)	598.0 (071.8)

S, sample; N, patient number. Samples T (tumor) and samples NT (non tumor) were collected as described and store at -80°C until assayed for SMO, APAO, ODC and SSAT activity, as described in Methods. Values are mean (SD).

### Active site modeling of human SMO-BENSpm and SMO-CPENSpm complexes

**Figure 3:**
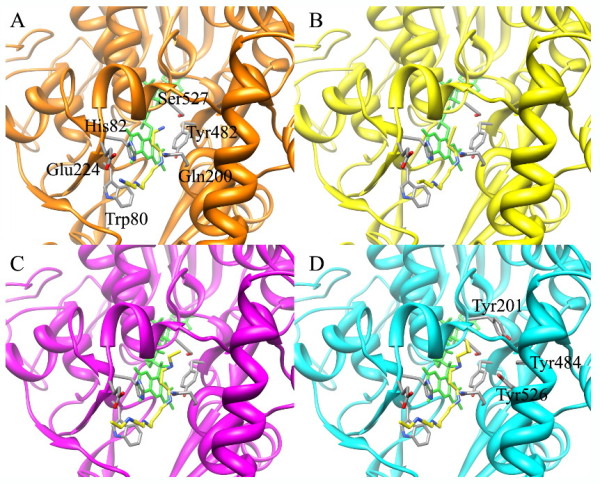
**Schematic representation of the putative complexes formed by mSMO with the substrate Spm (A), the inhibitor MDL 72527 (B), and the analogues BENSpm (C) and CPENSpm (D)**. For details see Methods section.

In order to analyze the potential inhibition properties of BENSpm and CPENSpm (Figure 3), a comparative structural analysis of the modeled complexes formed by mSMO with Spm, BENSpm, CPENSpm and the PAOs inhibitor MDL 72527 has been performed. Figure 3 shows the predicted interactions occurring between inhibitor/analogues chemical groups and mSMO active site residues. All three molecules analyzed are able to establish Spm-like interactions with residues His82, Gln200 and Tyr482. However, CPENSpm displays potential additional interactions since its cyclopropyl group fits well in a hydrophobic pocket formed by residues Tyr201, Tyr484 and Tyr526. This is expected to increase the affinity of the enzyme for CPENSpm as compared to both shorter MDL 72527 and BENSpm compounds that do not display the bulky hydrophobic cyclopropyl substituent.

### Inhibition of mSMO by Spm analogues BENSpm and CPENSpm

**Figure 4:**
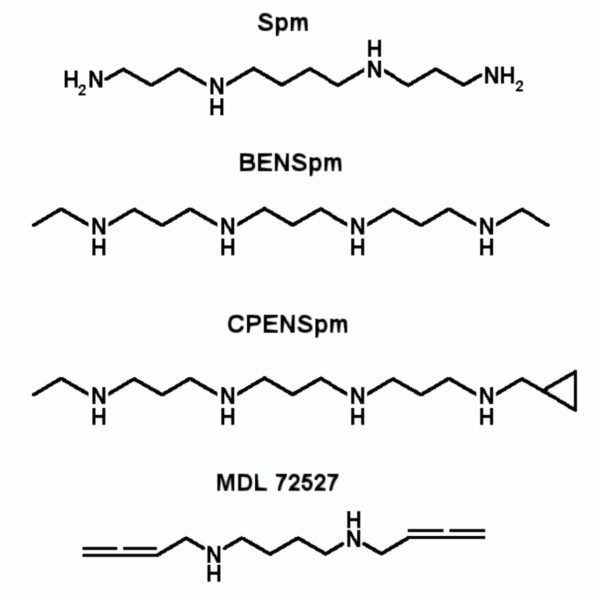
**Chemical structures of SMO substrates and inhibitors**. Chemical structures of Spm, the specific substrate of SMO enzyme, of MDL 72527 (N^1^, N^4^-bis(2,3-butadienyl)-1,4butanediamine), an inhibitor of SMO, and of Spm-derivative analogues BENSpm (N^1^, N^11^-di(ethyl)norspermine) and CPENSpm (N^1^-cyclopropyl-methyl-N^11^-ethyl-norspermine). Abbreviations used in the figure are defined in the text.

Murine recombinant SMO (mSMO) has been previously characterized [32]. This protein shares 95% amino acid sequence identity with the human counterpart and shows a 100% conservation of the residues involved in the catalytic properties [32]. The Spm analogues tested (Figure 4) as *in vitro *modulators of mSMO activity belong to two bis(alkyl)spermine classes: symmetrically substituted (BENSpm) and asymmetrically substituted (CPENSpm). Both molecules impair mSMO activity competitively. The inhibition of mSMO activity by BENSpm, CPENSpm and MDL72527 is shown in Table 3. Values of *K*_i _for mSMO competitive inhibition by these two Spm analogues are consistent with their predicted interaction mode observed in the modeled complexes. In fact, CPENSpm displays a much higher affinity for mSMO with respect to BENSpm and this is likely due to the cyclopropyl substituent present in CPENSpm which can be easily accommodated in the highly hydrophobic pocket formed by three Tyr residues with a total contact surface of approx. 73 Å^2^. This increase in hydrophobic contact surface area with respect to BENSpm is expected to improve the binding affinity of CPENSpm, according to known semiempirical expression of the relationship between contact surface and hydrophobic interactions energy [33], as indeed observed experimentally (Table 3).

**Table 3:** Values of *K*_i _for BENSpm, CPENSpm and MDL72527 binding to mSMO enzyme

Inhibitor	mSMO *K*_i _(M)^a^
BENSpm	3.8 × 10^-4^
CPENSpm	8.5 × 10^-5^
MDL72527	6.3 × 10^-5^

The SD is 5%.

## Discussion

This is the first investigation demonstrating that SMO expression in BC tissues is significantly lower than in nonneoplastic tissues, by RT-PCR and enzyme activity analyses.

It has been proposed that the H_2_O_2 _produced during the oxidation of PA by SMO may contribute to the level of apoptosis in BC cell lines [11,15]. Thus, it is tempting to speculate that the significant decreases in SMO activity that we observed in BC tissues may contribute to tumor growth through a decreased rate of endogenous apoptosis resulting from decreases in the local concentrations of H_2_O_2_. SMO dysregulation has also been observed in prostate cancer, by using image analysis techniques and TMAJ software tools [34], and in ulcerative colitis, by TaqMan-based real-time PCR [35]. In both cases the level of SMO expression was observed to be upregulated. To explain these apparently contradictory results of SMO expression the process of carcinogenesis has to be considered. It is well known that a large number of human cancer types has been directly associated to chronic inflammation, a temporally limited adaptive response. During inflammation there is an oxidative stress and many evidences point out that SMO activity participates in producing reactive oxygen species (H_2_O_2_) [6,36,37]. When the inflammation-driven tumour progressively develops, the H_2_O_2 _produced by oxidation of PA by SMO and APAO may potentially negatively contribute to cell proliferation. In this scenario, we expected to observe an inversion tendency of SMO and APAO gene expression, resulting in a lower ROS production that no longer contrasts the tumour progression.

In line with this hypothesis, we confirm that APAO enzyme activity was significantly lower in BC tissue [12], contributing to a reduction of the cellular H_2_O_2 _level. We also confirm the results of previous studies on SSAT and ODC enzyme activities carried out in human BC tissues compared with nonmalignant control, indicating a high ODC and SSAT activities in BC samples [12,38,39]. Manni et al. [38] reported that increased ODC activity is associated with an increased risk of both disease recurrence and death, while Deng et al. [39] showed that the overexpression of ODC in BC tissues correlates with the TNM grading system. Wallace et al. [12] reported that the decrease of APAO activity in BC tissues positively correlated with the aggressiveness of the tumor, while the increase of SSAT activity showed a tendency to be indicative of a poor prognosis. It was proposed that SSAT and APAO interplay could produce an efficient system to generate locally high concentrations of H_2_O_2 _that drives cells to the death signaling pathway, notwithstanding SMO activity was not considered in this context [12].

In BC cells this normal death-generating pathway is blocked by the decrease in activity of APAO and, thus, acetylPA accumulate within the tissue. AcetylPA, unlike PA, are not significantly toxic to tumor cells and may, thus, remain within the tumor cells without causing any negative effects [12]. On the other hand, the increase in SSAT activity explains the high level of N^1^, N^12^-diacetylspermine (DiAcSpm) in the urine of BC patients, which proved to be a more sensitive marker than CEA, CA19-9 and CA15-3 in BC at early stages [40].

Considering this altered PA homeostasis in BC tissue, a low gene expression of both SMO and APAO is in line with the tumoral blocked death-generating pathway and the high proliferating cellular rate. BENSpm and CPENSpm have been successfully employed as antiproliferate compounds on some human BC cell lines [11,15,17,18] but in Phases I and II of clinical trials gave poor positive outcomes [14,18,20-22]. The H_2_O_2 _produced through BENSpm-induced PA catabolism was found to be derived exclusively from SMO and not through APAO activity. This data suggested that SMO activity is the major mediator of the cellular response of BC cells to BENSpm and that APAO plays little or no role in this response [11]. It has been shown that the utilization of CPENSpm has produced overlapping results with the BENSpm treatments [22]. We modeled the structure of the complexes formed by the mSMO enzyme with BENSpm and CPENSpm. This modeling analysis, a fast and economic way of screening a large number of potential Spm analogues, has revealed the structural bases of the higher affinity that CPENSpm displays for mSMO active site with respect to BENSpm. The cyclopropyl substituent present in CPENSpm nicely fits in a hydrophobic pocket present in mSMO active site. This interaction, estimated to increase CPENSpm affinity for mSMO, is absent in the modeled BENSpm-mSMO. To confirm that these molecules impair mSMO activity, the inhibition of this enzyme activity by BENSpm and CPENSpm was analyzed. Values of *K*_i _for mSMO competitive inhibition by these two Spm analogues indicate that they can be considered good *in vitro *inhibitors of SMO activity, with CPENSpm being more reactive than BENSpm. These results are apparently in contradiction with previous data reported by Wang et al. [41], indicating that both BENSpm and CPENSpm molecules are poor inhibitors of the human SMO enzyme. However, in that work the authors were searching for strong SMO inhibitors and used a very low inhibitor concentration (10 μM) compared to substrate concentration (250 μM). The high Spm concentration utilized in Wang's experiments would explain the poor inhibition observed [41]. In our experimental conditions the BENSpm and CPENSpm concentration was in the range of 10^-4 ^M in the presence of 5-10 × 10^-6 ^M Spm. BENSpm and CPENSpm show *K*_i _values (3.8 × 10^-4 ^M and 8.5 × 10^-5 ^M, respectively) comparable to that of MDL72527 (6.3 × 10^-5 ^M), which can be considered a good inhibitor of SMO activity [42]. The finding that both BENSpm and CPENSpm are inhibitors of the SMO catalytic activity could explain their SMO induction effect as a cellular mechanism to overwhelm enzyme inhibition. This novel finding on the inhibitory properties of BENSpm and CPENSpm should not be underestimated and could explain the clinical trials failure of BENSpm. Nevertheless, a key question is to understand how intracellular SMO and SSAT up-regulations are exerted by BENSpm and CPENSpm treatment. One hypothesis is that these analogues compete with natural PA for uptake when using the PA transporter to gain entry into the cell [5].

## Conclusions

Data in this article highlight the clinical importance of SMO expression in breast tumors. SMO enzyme is an important PA catabolic component that could play a crucial role in BC disease and still remains a promising therapeutic target for cancer and hyperproliferation. This is the first study demonstrating that SMO activity in BC tissues is significantly lower than in controls. Low SMO activity may contribute to tumor growth through a decrease of the local H_2_O_2 _concentration. Up-regulation of SMO activity can lead to an increase of apoptosis rate. Spm analogues able to induce SMO activity can be utilized in anticancer therapy. Two Spm analogues, BENSpm and CPENSpm, capable of producing cytotoxicity on some human BC cell lines by SMO induction, have been *in silico *analyzed and then *in vitro *tested. Both analogues resulted to be inhibitors of SMO activity. Their inhibition properties could explain their failure in clinical trials. The *in silico *Spm analogues screening together with the availability of the recombinant SMO enzyme could be relevant to *in vitr*o test analogues able to up-regulate SMO, before their utilization in clinical trials. Hopefully, new selected Spm analogues could be utilized as antineoplastic drugs with novel action mechanisms.

## List of abbreviations

APAO: *N*^1^-acetylpolyamine oxidase; BC: breast cancer; BENSpm: bis(ethyl)norspermine; CPENSpm: (cyclopropyl)-methyl-4,8-diazaundecane; GADPH: glyceraldehyde-3-phosphate dehydrogenase; MDL 72527: (N^1^, N^4^-bis(2,3-butadienyl)-1,4butanediamine); NT: nontumor; ODC: ornithine decarboxylase; PLS-DA: partial least squares-discriminant analysis; SD: Standard Deviation; SSAT: spermidine/spermine *N*^1^-acetyltransferase; SMO: spermine oxidase; T: tumor; TNM: tumor size grading system.

## Competing interests

The authors declare that they have no competing interests.

## Authors' contributions

PM, RF and RA designed and coordinated research. FS, GG, RG provided clinical samples. EF, GB and MC a performed experimental research. FP and MB performed modeling and statistical analyses. RAC conceived the study and helped to draft the manuscript. PMW performed the synthesis of Spm analogues. All authors read and approved the manuscript.

## Pre-publication history

The pre-publication history for this paper can be accessed here:

http://www.biomedcentral.com/1471-2407/10/555/prepub

## Supplementary Material

Additional file 1 **Table S1**. Primers used in this study. Click here for file

Additional file 2 **Table S2**. Enzyme activities in human BC tissue samples. Click here for file
