# Supplementary material for: Spermine oxidase (SMO) activity in breast tumor tissues and biochemical analysis of the anticancer spermine analogues BENSpm and CPENSpm
Source: BMC Cancer. 2010 Oct 14;10:555. doi: 10.1186/1471-2407-10-555 (PMC3027604; doi:10.1186/1471-2407-10-555)
Supplement: Additional file 1 — Table S1. Primers used in this study. [file 1471-2407-10-555-S1.doc]

**Additional file 1**

| **Target gene** | **Name** | **Primer sequence** |
| --- | --- | --- |
| SMO | SMO1-F | 5'-AGGCCTGCCCACAGAGAAGG-3’ |
| SMO | SMO1-R | 5'-TGCATGGGCGCTGTCTTTG-3’ |
| APAO | APAO-F | 5’-GGACGCCTGGTTCCGGAAG-3’ |
| APAO | APAO-R | 5’-TGCGATGTGTGGCTTCCCC-3’ |
| ODC | ODC-F | 5’-TTGGAACGGGCGAAAGAGC-3’ |
| ODC | ODC-R | 5’-CATAAAGGTCTGCTCACTCG-3’ |
| SSAT | SSAT-F | 5’-CAGCCACTGCCGCCGACT-3’ |
| SSAT | SSAT-R | 5’-GCAAGTACTCCTTGTCGATC-3’ |
| GADPH | GADPH-F | 5’-CCATGGAGAAGGCTGGGG-3’ |
| G ADPH | GADPH-R | 5’-CAAAGTTGTCATGGATGACC-3’ |
| β-ACTIN | ACT-F | 5’-AAGAGAGGCATCCTCACCCT-3’ |
| β-ACTIN | ACT-R | 5’-TACATGGCTGGGGTGTTGAA-3’ |

**Table S1.** Primers used in this study

Target gene acronyms are defined in Methods.
