# Supplementary material for: Spermine oxidase (SMO) activity in breast tumor tissues and biochemical analysis of the anticancer spermine analogues BENSpm and CPENSpm
Source: BMC Cancer. 2010 Oct 14;10:555. doi: 10.1186/1471-2407-10-555 (PMC3027604; doi:10.1186/1471-2407-10-555)
Supplement: Additional file 2 — Table S2. Enzyme activities in human BC tissue samples. [file 1471-2407-10-555-S2.doc]

**Additional file 2**

**Table S2.** Enzyme activities in human BC tissue samples

| **Sample** | **SMO activity**  (pmol H_2_O_2_/  mg protein/h) | **APAO activity**  (pmol H_2_O_2_/  mg protein/h) | **ODC activity**  (pmol CO_2_/  mg protein/h) | **SSAT activity**  (pmol CO_2_/  mg protein/h) |
| --- | --- | --- | --- | --- |
| T1 | 364.01 (10.8) ) | 317.09 (14.1) | 117.7 (5.0) | 933.0 (25.7) |
| NT1 | 731.15 (11.9) ) | 893.19 (44.9) | 7.7 (1.1) | 555.8 (39.6) |
| T2 | 384.75 (06.2) ) | 435.14 (10.9) | 149.7 (3.8) | 1,023.6 (107.0) |
| NT2 | 863.78 (12.9) ) | 727.93 (22.6) | 12.8 (0.6) | 703.5 (11.9) |
| T3 | 219.45 (11.7) ) | 413.29 (12.4) | 650.6 (9.4) | 3,023.4 (68.6) |
| NT3 | 860.13 (130.0) | 3,101.36 (131.9) | 17.4 (1.9) | 1,316.4 (121.9) |
| T4 | 390.91 (11.6) ) | 472.16 (14.0) | 79.6 (4.5) | 1,923.1 (69.0) |
| NT4 | 3,500.50 (172.1) | 731.15 (13.5) ) | 19.3 (1.2) | 897.6 (45.2) |
| T5 | 136.66 (8.9) | 81.04 (14.3) | 31.4 (1.3) | 1,133.5 (43.3) |
| NT5 | 338.28 (7.7) | 185.13 (31.8) ) | 8.1 (0.3) | 727.3 (36.2) |
| T6 | 135.64 (9.9) | 102.28 (15.1) ) | 138.6 (7.3) | 147.5 (22.9) |
| NT6 | 206.83(11.1) | 395.06 (123.7) | 3.5 (0.3) | 55.9 (14.7) |
| T7 | 140.70 (14.2) ) | 112.90 (15.1) ) | 280.3 (4.7) | 3,635.5 (22.4) |
| NT7 | 452.55(13.7) ) | 452.26 (33.7) ) | 3.4 (0.3) | 222.1 (28.6) |
| T8 | 129.58 (9.9) | 108.85 (15.1) | 57.0 (3.4) | 1,450.6 (102.9) |
| NT8 | 328.22 (21.6) | 498.37 (34.9) ) | 7.5 (0.9) | 838.8 (99.3) |
| T9 | 69.13 (5.4) | 70.59 (23.4) | 77.1 (1.9) | 1,964.0 (15.6) |
| NT9 | 918.75 (18.7) | 173.00 (13.7) ) | 9.6 (2.7) | 378.3 (12.7) |
| T10 | 70.96 (8.8) | 71.32 (29.5) ) | 19.3 (0.4) | 3,190.6 (190.8) |
| NT10 | 693.00 (19.7) | 249.44 (53.7) ) | 7.7 (0.3) | 474.0 (26.9) |
| T11 | 65.47 (7.8) | 64.37 (09.5) | 119.9 (1.8) | 6,337.1 (590.5) |
| NT11 | 133.13 (32.2 ) | 288.94 (44.7) ) | 6.7 (0.5) | 239.0 (25.3) |
| T12 | 89.97 (13.4) ) | 51.57 (15.1) ) | 157.7 (2.1) | 2,007.3 (36.4) |
| NT12 | 560.00 (22.4) ) | 296.99 (38.7) | 8.4 (1.4) | 164.9 (29.7) |
| T13 | 61.45 (11.5) ) | 76.81 (35.4) | 74.3 (0.7) | 1,759.1 (87.4) |
| NT13 | 148.49 (26.7) ) | 179.95 (73.1) | 13.0 (0.3) | 770.9 (43.1) |
| T14 | 68.76 (15.3) | 55.59 (43.8) | 52.0 (3.3) | 643.0 (21.3) |
| NT14 | 94.73 (9.5) | 150.69 (72.8) ) | 9.4 (1.5) | 456.1 (11.8) |
| T15 | 95.09 (10.6) ) | 54.86 (34.7) | 581.5 (15.2) | 678.6 (24.7) |
| NT15 | 288.57 (19.9) ) | 332.46 (37.3) ) | 11.1 (2.8) | 237.5 (23.0) |
| T16 | 265.55 (17.1) | 913.29 (14.4) ) | 876.6 (9.4) | 3,231.5 (210.2) |
| NT16 | 860.13 (31.8) ) | 2,101.36 (207.5) | 23.4 (5.9) | 861.7 (81.6) |
| T17 | 224.55 (12.9) ) | 108.85 (22.1) ) | 275.7 (9.4) | 1,740.6 (72.3) |
| NT17 | 528.22 (32.6) | 307.73 (32.5) ) | 31.5 (2.9) | 900.8 (66.9) |
| T18 | 123.96 (23.8) | 101.32 (11.5) | 419.3 (20.4) | 4,870.0 (365.8) |
| NT18 | 654.26 (32.7) | 432.44 (35.8) | 57.8 (8.3) | 798.7 (36.2) |
| T19 | 101.79 (13.4) ) | 153.97 (43.1) | 516.5 (6.2) | 2,320.3 (63.8) |
| NT19 | 560.76 (43.0) ) | 962.04 (87.3) | 22.4 (3.4) | 846.0 (54.2) |
| T20 | 56.79 (21.7) ) | 49.78 (15.1) | 215.3 (5.1) | 980.3 (36.4) |
| NT20 | 342.00 (42.4) | 168.24 (21.5) | 98.4 (1.4) | 514.9 (15.9) |

Values are mean (SD) of three independent experiments performed in duplicate. The p values measured by the Student’s t test were 0.02, 0.04, 0.003 and 0.001 for SMO, APAO, ODC and SSAT, respectively. Samples T (tumor) and samples NT (non tumor) were collected as described and store at -80°C until assayed for SMO, APAO, ODC and SSAT activity, as described in Methods.
